# Supplementary material for: Prion protein lowering is a disease-modifying therapy across prion disease stages, strains and endpoints
Source: Nucleic Acids Res. 2020 Aug 10;48(19):10615–31. doi: 10.1093/nar/gkaa616 (PMC7641729; doi:10.1093/nar/gkaa616)
Supplement: gkaa616_Supplemental_File [file gkaa616_supplemental_file.pdf]

SUPPLEMENTARY MATERIALS

Prion protein lowering is a disease-modifying therapy across prion strains, disease stages, and endpoints

Eric Vallabh Minikel[1,2,3,4,5], Hien T Zhao[6], Jason Le[7], Jill O'Moore[8], Rose Pitstick[8], Samantha Graffam[7], George A Carlson[8], Jasna Kriz[9], Jae Beom Kim[10], Jiyan Ma[11], Holger Wille[12], Judd Aiken[12], Deborah McKenzie[12], Katsumi Doh-ura[13], Matthew Beck[7], Rhonda O'Keefe[7], Jacquelyn Stathopoulos[7], Tyler Caron[7], Stuart L Schreiber[7,14], Jeffrey B Carroll[15], Holly B Kordasiewicz[6,†], Deborah E Cabin[8,†], Sonia M Vallabh[1,2,3,4,5,†]

1. Stanley Center for Psychiatric Research, Broad Institute of MIT and Harvard, Cambridge, MA, 02142, USA
2. Prion Alliance, Cambridge, MA, 02139, USA
3. Henry and Allison McCance Center for Brain Health, Massachusetts General Hospital, Boston, MA, 02114, USA
4. Department of Neurology, Massachusetts General Hospital, Boston, MA, 02114, USA
5. Harvard Medical School, Boston, MA, 02115, USA
6. Ionis Pharmaceuticals Inc, Carlsbad, CA, 92010, USA
7. Broad Institute of MIT and Harvard, Cambridge, MA, 02142, USA
8. McLaughlin Research Institute, Great Falls, MT, 59405, USA
9. Cervo Brain Research Center, Université Laval, Québec, QC, G1J 2G3, Canada
10. PerkinElmer, Hopkinton, MA, 01748, USA
11. Center for Neurodegenerative Science, Van Andel Institute, Grand Rapids, MI, 49503, USA
12. University of Alberta, Edmonton, AB, T6G 2M8, Canada
13. Department of Neurochemistry, Tohoku University Graduate School of Medicine, Sendai, Miyagi, 980-8575, Japan
14. Department of Chemistry & Chemical Biology, Harvard University, Cambridge, MA, 02138, USA
15. Western Washington University, Bellingham, WA, 98225, USA

†To whom correspondence should be addressed: svallabh@broadinstitute.org, deborahcabin@mclaughlinresearch.org, or hkordasiewicz@ionisph.com

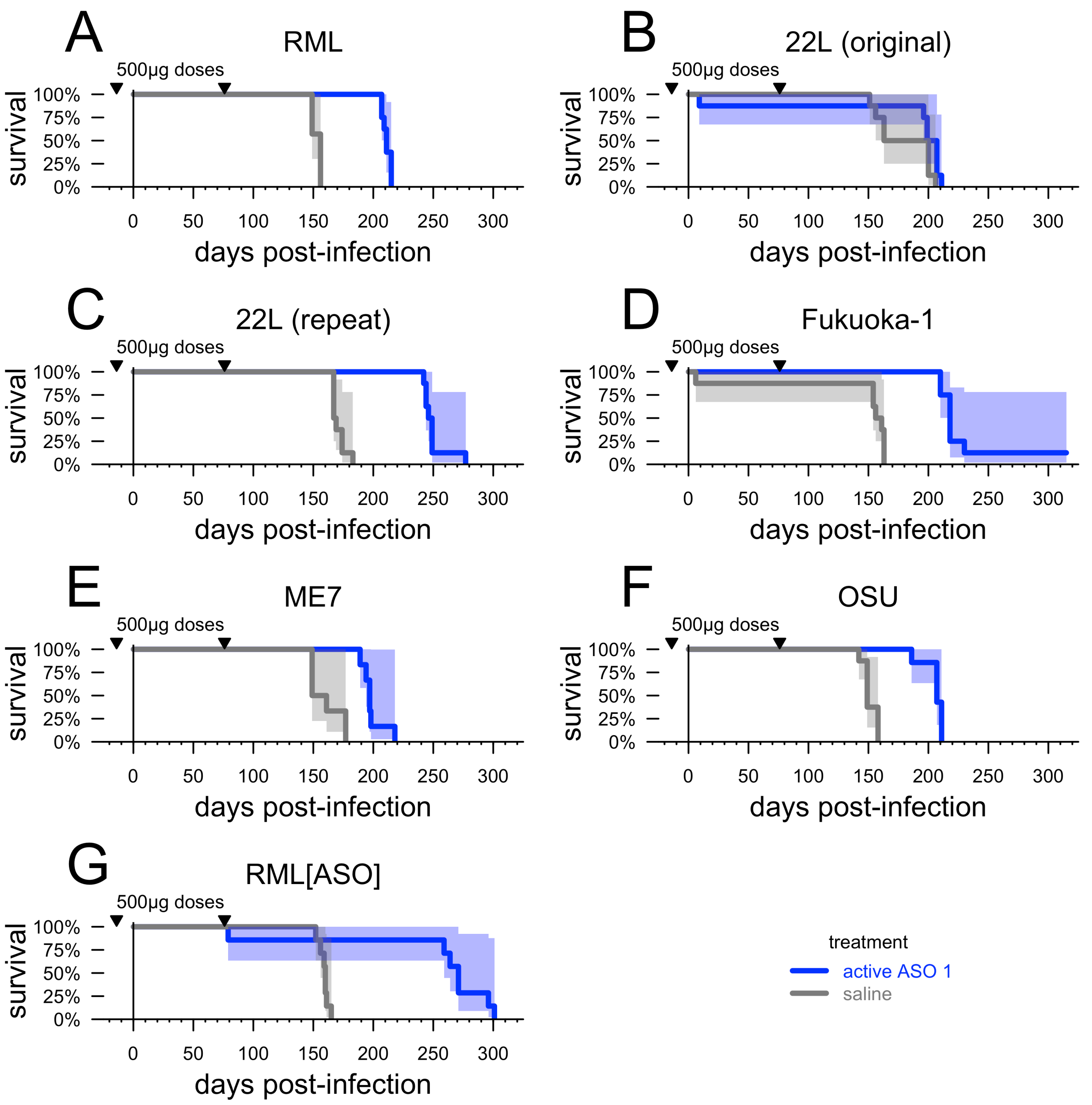

Figure S1. Survival curves for prophylactically ASO-treated animals across prion strains. *Data summarized in Table 2. Shaded areas represent 95% confidence intervals. In the original 22L experiment (B), we observed only a marginal increase in survival time in ASO-treated animals (+12%, P = 0.09, two-sided log-rank test). We suspected an experimental error because the distribution of survival times was bimodal among control animals: 4/4 saline-treated animals in one cage succumbed at 158±6 dpi, while 4/4 saline-treated animals in the other cage succumbed at 202±3 dpi. This latter cage had been labeled "E" while an adjacent active ASO-treated cage had been labeled "F", leading us to suspect the two cage cards had been swapped and that some of the 22L control animals had in fact received one dose of ASO. We repeated the experiment, again with blinded veterinary technicians performing all animal evaluations, and obtained the result in panel (C), which is summarized in Table 2.*

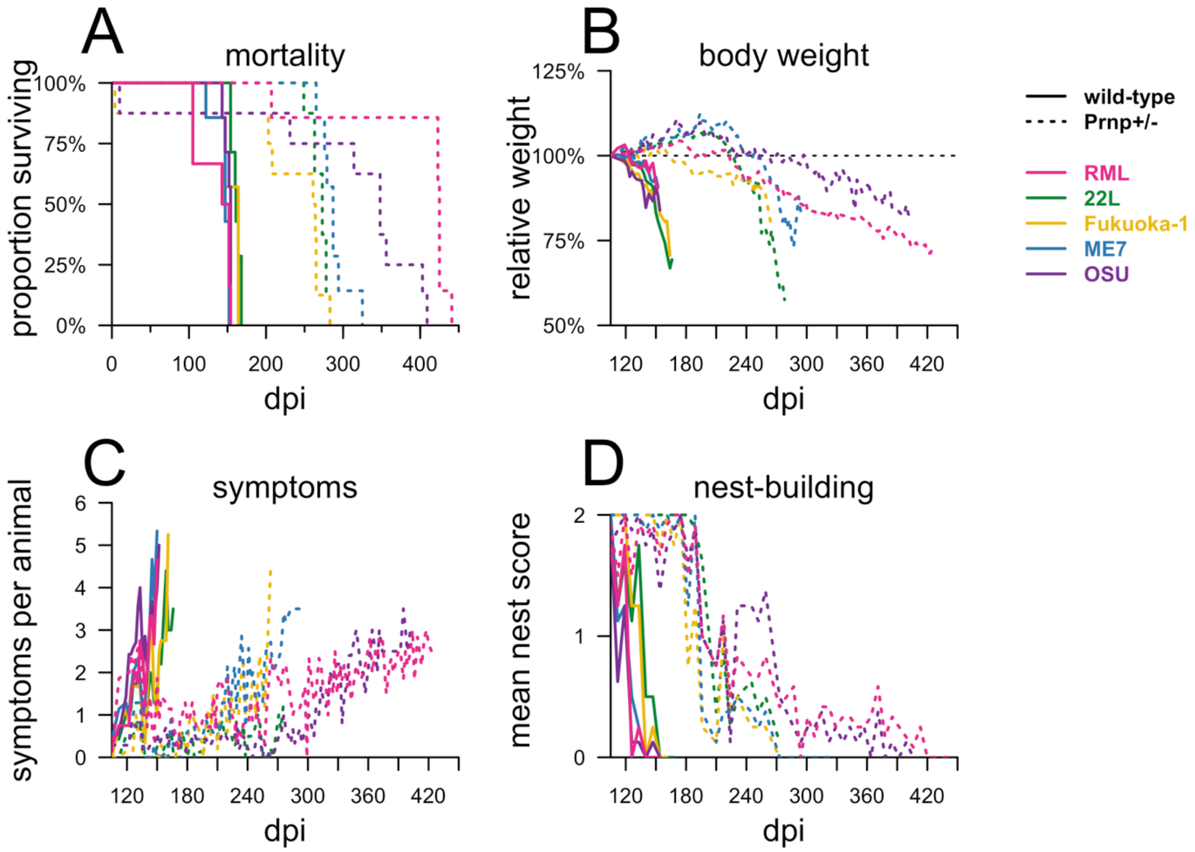

Figure S2. Endpoints in heterozygous PrP knockout mice infected with five prion strains. *Details on animals summarized in the right half of Table 2.* ***A)*** *survival,* ***B)*** *body weights relative to 105 dpi baselines (relative rather than absolute weights are used because the cohorts contain different proportions of male and female mice),* ***C)*** *mean symptom count per animal, and* ***D)*** *mean nest score.*

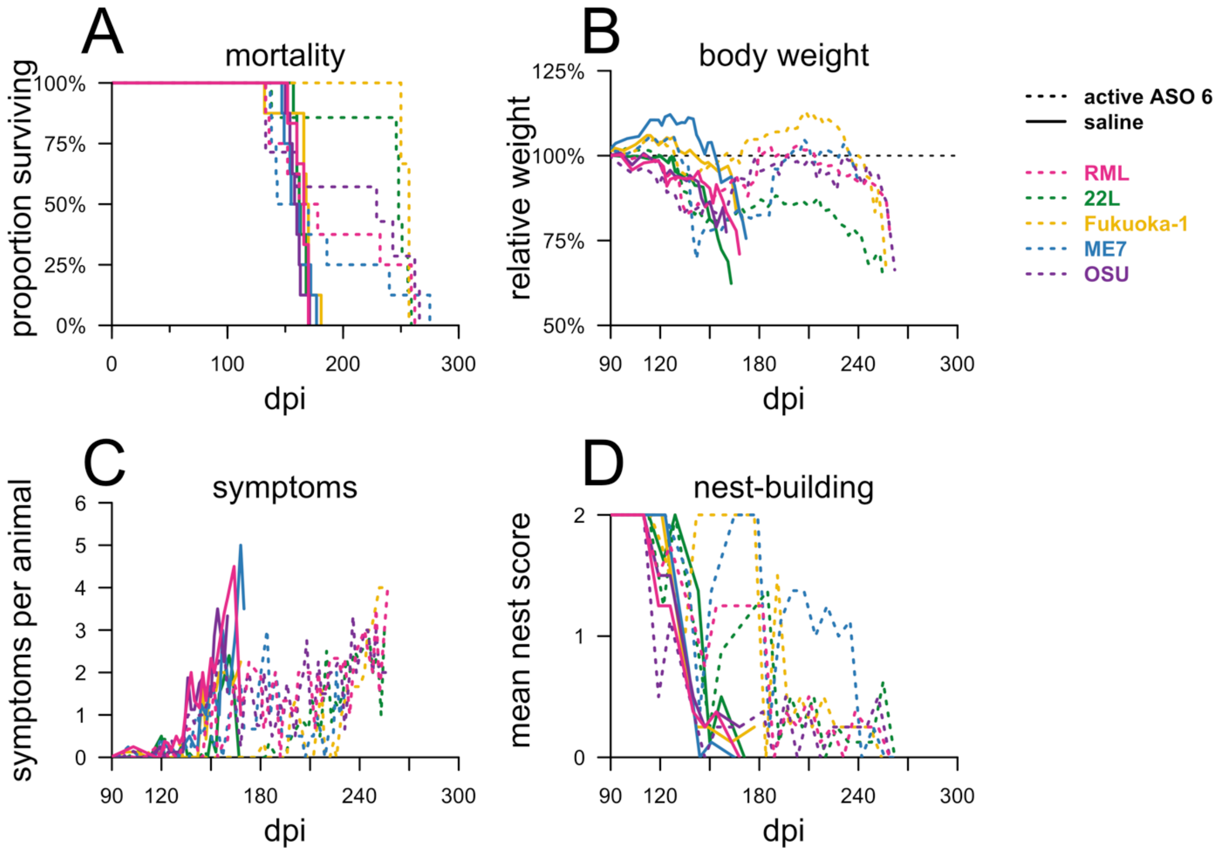

Figure S3. Endpoints in mice infected with five prion strains receiving late ASO or saline treatment. *Details on animals summarized in Table 3.* ***A)*** *survival,* ***B)*** *body weights relative to 105 dpi baselines,* ***C)*** *mean symptom count per animal, and* ***D)*** *mean nest score.*

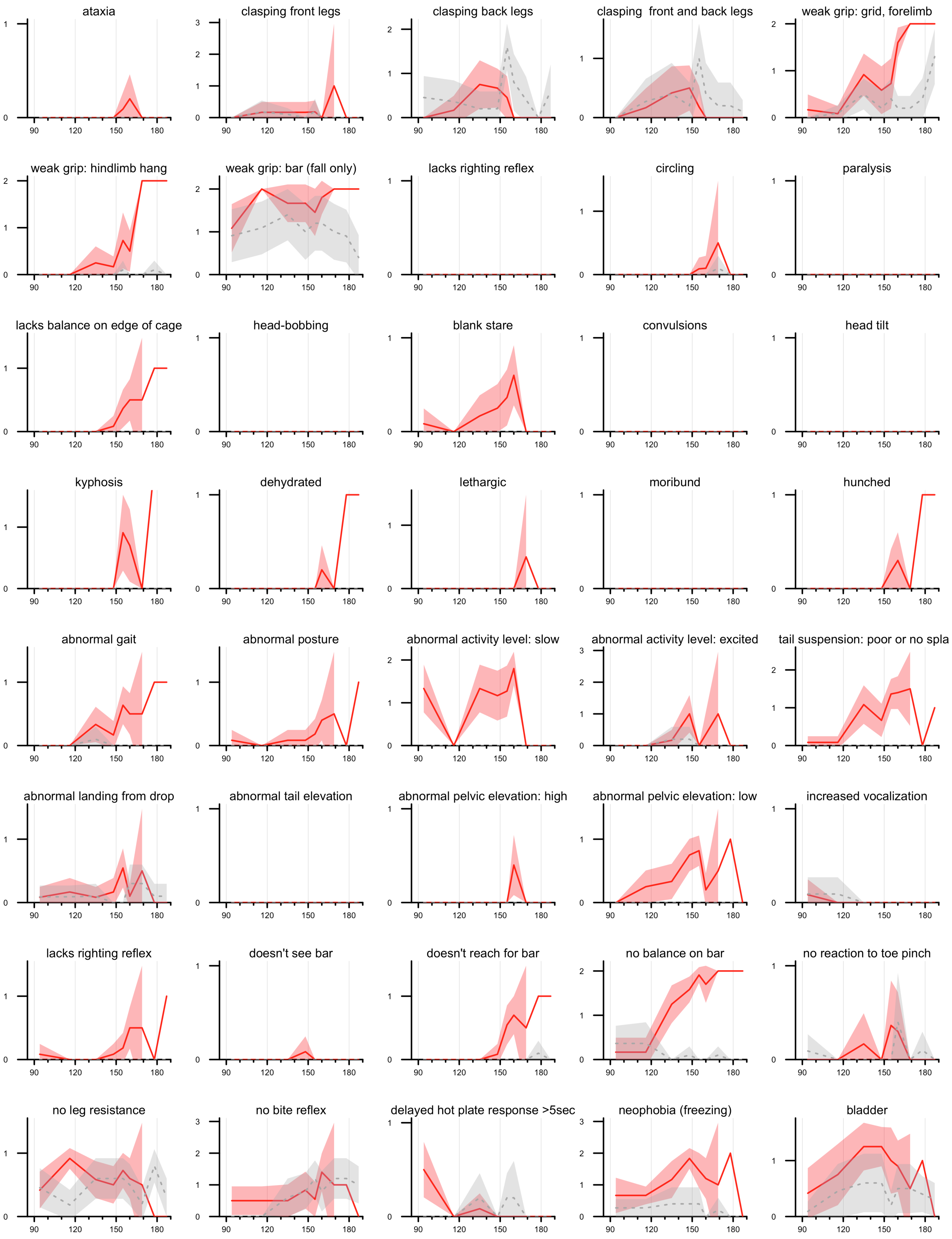

Figure S4. Behavioral observations in the natural history of RML prion infection. *Data from Figure 4C broken into all N=40 Individual behavioral observations (listed in Table S2).*

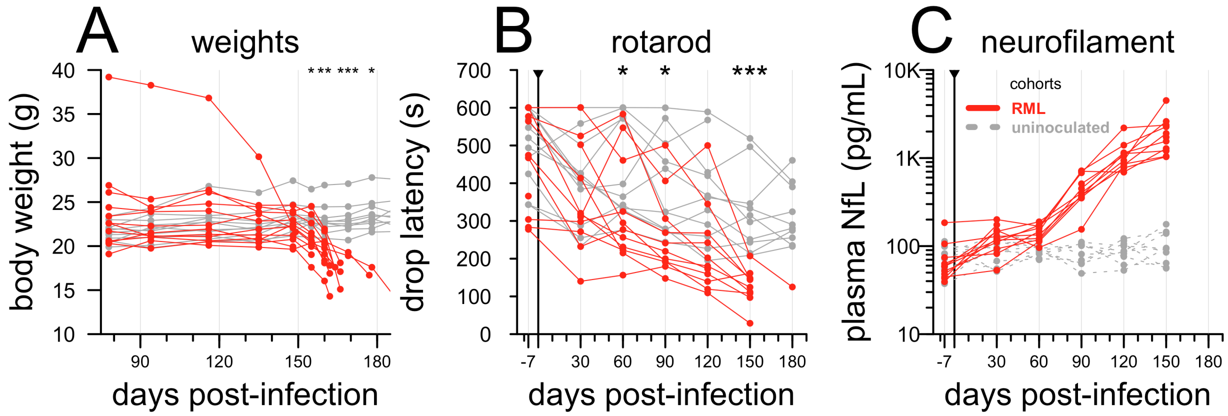

Figure S5. Raw individual natural history endpoints. *Alternative visualizations of data from Figure 4:* ***A)*** *individual body weights,* ***B)*** *individual mean rotarod latency, and* ***C)*** *individual plasma NfL concentrations (note log y axis).*

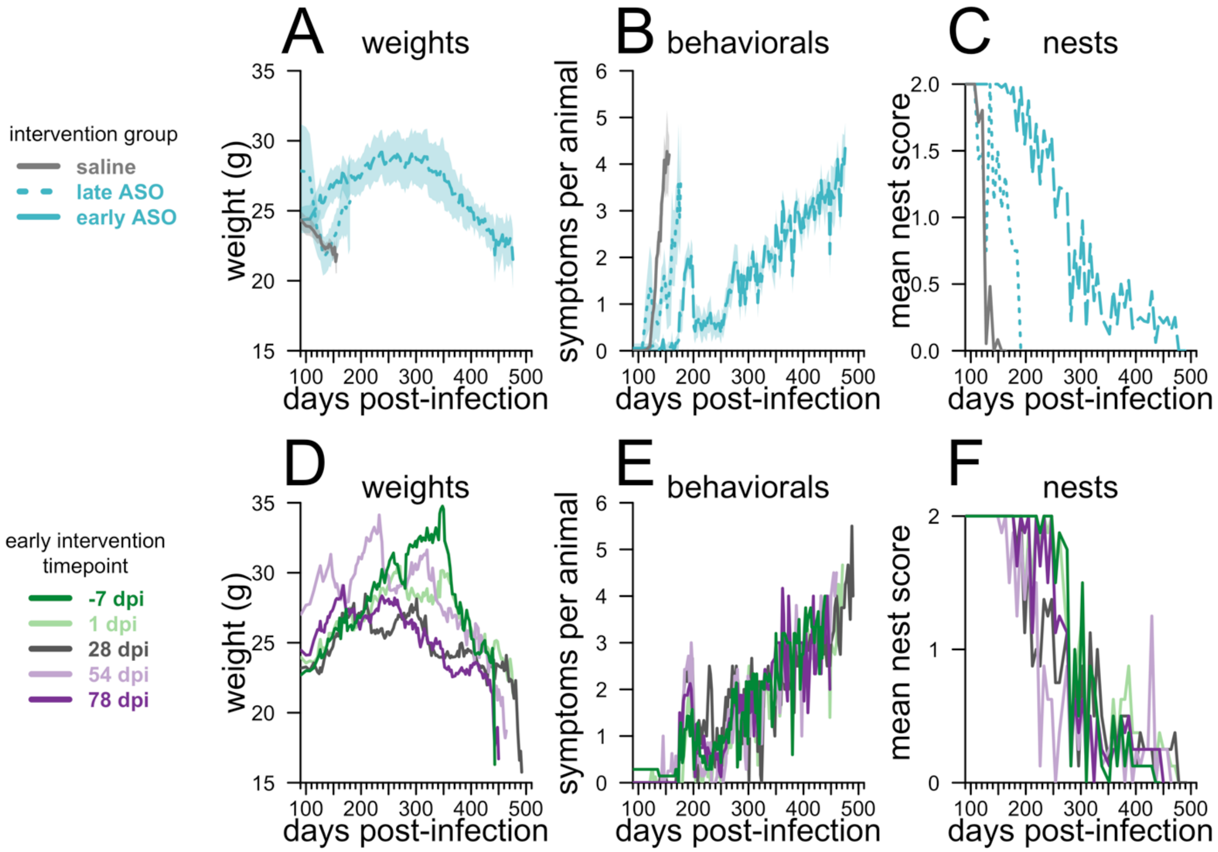

Figure S6. Endpoints in mice receiving chronic ASO or saline treatment beginning at different timepoints. *Details on animals summarized in Figure 6: weights (**A,D**), mean symptom count per animal (**B,E**), and nest score (**C,F**). **A-C**) animals are grouped by saline or early (-7 to 78 dpi) versus late (105 and 120 dpi) ASO treatment initiation, as in Figure 6B. **D-F**) early intervention (-7 to 78 dpi) animals are grouped by individual intervention timepoint.*

Table S1. Detailed criteria and performance statistics for behavioral tests. *Building on commonly used neurological evaluations in the prion field[2], we initially (experiments reported in Raymond et al[3] and in Figure 2D-F) evaluated animals for six neurological signs: tremor, ataxia, difficulty righting, tail rigidity, blank stare, and hindlimb weakness. Based on those initial experiences, after removing signs found non-contributory, grouping those found redundant, and adding signs sometimes noted in comments, our behavioral battery was revised to the above list for all other experiments at the Broad Institute reported herein (Figure 2A-C, 3, 6-7, and Tables 2-3). Raters were asked to assess each animal individually for 15 seconds on a cage lid and to record only observations made during this period. Each sign was then recorded as a 1 or 0 in a blank set of spreadsheet columns. Specific criteria or guidance were provided to raters for a subset of tests and are displayed above; other tests were never described in greater detail verbally but were conveyed through hands-on training. In order to assess the baseline performance characteristics of each test, we retrospectively analyzed N=119 saline-treated, RML prion-infected control animals across all experiments. For each animal, time of onset of each symptom was defined as the first timepoint where the symptom was i) observed at that timepoint, and ii) observed in at least half of subsequent observations of that animal. Displayed are the mean±sd of the lead time (time from onset of specific symptom to prion disease endpoint) and the proportion of animals in which the symptom was ever observed.*

behavioral test	criteria/guidance	lead time to disease endpoint (mean±sd)	proportion of animals ever showing symptom
tremor	continuous tremor visible to the naked eye (periodic tremors, or tremors that can be felt but not seen, do not count)	20.1 ± 10.2	98%
reduced activity	moves <50% as much or <50% as fast as a normal mouse	18.8 ± 8.3	97%
scruff/poor grooming	full body scruff (neck only does not count); mice with boosts cannot be scored	8.7 ± 6.7	86%
irregular gait/hindlimb weakness	—	8.0 ± 5.1	73%
blank stare	—	4.3 ± 3.1	55%
hunched posture	—	4.0 ± 3.8	40%
poor body condition	body condition score[1] ≤2	2.7 ± 1.5	5%
difficulty righting	perturb the mouse by lying on its side and see if it stands up again	2.6 ± 1.5	8%

Table S2. Endpoint data for animals in Figures 2-5 in tabular form. *Only animals that reached pre-specified euthanasia endpoint are included here. For overall mortality data see survival curves in the main figures or the online data repository described in Methods. *In the uninoculated group, no animals developed disease; the experiment was terminated at 190 dpi.*

**Experiment**	**cohort**	**mean±sd**	***N***	**Δ**
MOE ASO prophylactic (Figure 2A-C)	saline	150.7 ± 5.5	9	—
	control ASO 4	157.9 ± 4.9	10	+5%
	active ASO 5	304.8 ± 19.9	6	+102%
	active ASO 6	276.8 ± 11.6	9	+84%
MOE ASO 120 dpi (Figure 2D-F)	saline	161.7 ± 7.0	9	—
	control ASO 4	160.2 ± 11.0	9	-1%
	active ASO 5	139.5 ± 5.3	4	-14%
	active ASO 6	275.2 ± 3.2	8	+70%
Dose response (Figure 3)	saline	152.0 ± 5.3	8	—
	30 µg	173.6 ± 7.7	7	+14%
	100 µg	194.0 ± 6.7	6	+28%
	300 µg	207.2 ± 4.3	6	+36%
	500 µg	226.4 ± 14.7	7	+49%
Natural history (Figure 4)	uninoculated	*190**	0	—
	RML	164.8 ± 9.6	12	—
NfL (Figure 5A-B)	saline	160.7 ± 9.9	10	0%
	active ASO 6	253.0 ± 11.7	8	57%
BLI (Figure 5C-D)	saline	156.2 ± 12.1	8	0%
	control ASO 3	155.7 ± 9.8	3	0%
	active ASO 2	189	1	+21%
	active ASO 1	234.7 ± 18.1	7	+50%

Table S3. Measures observed in the natural history study.

category	observations	scoring
neurological	ataxia	1: difficulty getting across cage and /or falling over
neurological	clasping front legs	1: almost 2: full clasp
neurological	clasping back legs	1: almost 2: full clasp
neurological	clasping front and back legs	1: almost 2: full clasp
neurological	weak grip: grid, forelimb	1: weak or moderate 2: absent
neurological	weak grip: hindlimb hang	1: moderate (fall 1 out of 2 times) 2: weak fall 2 out of 2 tries (2x)
neurological	weak grip: bar (fall only)	1: moderate (fall 1 out of 2 times) 2: weak fall 2 out of 2 tries (2x)
neurological	lacks righting reflex (flipped on back by tail, not contact right reflex)	1: poor: all but head will roll over when tail is twisted. 2: lacking: can't right
neurological	circling	1: present
neurological	paralysis	1: paresis (limited range of motion for walking only, curled paw may be present, one limb non-weight bearing) 2: paralysis (cannot move)
neurological	lacks balance on edge of cage	1: present
neurological	head-bobbing	1: present
neurological	blank stare	1: present at initial box evaluation
neurological	convulsions	1: present
neurological	head tilt	1: present
neurological	kyphosis	1: slight to moderate 2: severe
non-neurological	dehydrated	1: present when nape pinched and/or sunken eyes
non-neurological	lethargic	1: present (immobile even after poked by finger)
non-neurological	moribund	1: present (near death)
non-neurological	hunched	1: present, not kyphosis, front and hind feet are close together when standing, not applicable to resting posture
SHIRPA	abnormal gait	1: wobble due to wide hind limb stance
SHIRPA	abnormal posture	1: present
SHIRPA	abnormal activity level: slow	1: present in home box only 2: no exploring in test box
SHIRPA	abnormal activity level: excited	1: moderate (noticed in home box or behavior box) 2: severe, may include jumping
SHIRPA	tail suspension: poor or no splay	1: poor 2: none or abnormally wide

SHIRPA	abnormal landing from drop	1: falls on tail or back from bar only (not drop from tail suspension)
SHIRPA	abnormal tail elevation	1: present
SHIRPA	abnormal pelvic elevation: high	1: present
SHIRPA	abnormal pelvic elevation: low	1: present
SHIRPA	increased vocalization	1: present
SHIRPA	lacks righting reflex	1: lacking
SHIRPA	doesn't see bar	1: present
SHIRPA	doesn't reach for bar	1: present
SHIRPA	no balance on bar	1: moderate: can balance after multiple tries or can only balance a second or two. 2: cannot balance
SHIRPA	no reaction to toe pinch	1: flinch 2: no reaction (zero: pulls paw away immediately)
SHIRPA	no leg resistance	1: doesn't react to pressure against paws (zero if any paw pushes back)
SHIRPA	no bite reflex	1: poor: notices but doesn't bite 2: no reaction (normal is immediate bite)
SHIRPA	delayed hot plate response >5sec	1: longer than 5 seconds
SHIRPA	neophobia (freezing)	1: any episode of freezing in test box (vs home box) 2: paralyzing fear
SHIRPA	bladder	0: not palpable (np) or good abdominal tone (gt), 1: small or medium 2: large or huge.

Table S4. Endpoint times by treatment timepoint in chronic dosing study. *Data from Figure 6 presented in tabular format. In contrast to the Results text, this presentation of the data uses mean±sd as opposed to median and includes only animals reaching pre-specified euthanasia endpoint. For overall mortality data see survival curves in the main figures or the online data repository described in Methods.*

	saline		active ASO 6		
**timepoint**	**survival dpi (mean±sd)**	***N***	**survival dpi (mean±sd)**	***N***	**Δ**
**-7**	143±4	7	394±77	4	+175%
**1**	145±3	8	462±18	6	+219%
**28**	145±11	6	426±109	7	+194%
**54**	150±5	8	377±124	5	+151%
**78**	149±7	7	406±47	4	+172%
**105**	151±4	8	184±5	5	+21%
**120**	152±7	8	172±8	7	+13%

Table S5. Survival times by treatment timepoint in symptomatic intervention study. *Data from Figure 7 presented in tabular format. In contrast to the Results text, this presentation of the data uses mean±sd as opposed to median and includes only animals reaching pre-specified euthanasia endpoint. For overall mortality data see survival curves in the main figures or the online data repository described in Methods.*

	saline		active ASO 6		
**timepoint**	**survival dpi (mean±sd)**	***N***	**survival dpi (mean±sd)**	***N***	**Δ**
**120**	167±9	12	243±50	7	+46%
**132**	164±14	8	187±57	8	+14%
**143**	168±7	12	207±65	7	+23%
**156**	165±5	5	163±5	7	-1%

SUPPLEMENTAL REFERENCES

1. Ullman-Culleré MH, Foltz CJ. Body condition scoring: a rapid and accurate method for assessing health status in mice. Lab Anim Sci. 1999 Jun;49(3):319–323. PMID: 10403450

2. Carlson GA, Kingsbury DT, Goodman PA, Coleman S, Marshall ST, DeArmond S, Westaway D, Prusiner SB. Linkage of prion protein and scrapie incubation time genes. Cell. 1986 Aug 15;46(4):503–511. PMID: 3015416

3. Raymond GJ, Zhao HT, Race B, Raymond LD, Williams K, Swayze EE, Graffam S, Le J, Caron T, Stathopoulos J, O'Keefe R, Lubke LL, Reidenbach AG, Kraus A, Schreiber SL, Mazur C, Cabin DE, Carroll JB, Minikel EV, Kordasiewicz H, Caughey B, Vallabh SM. Antisense oligonucleotides extend survival of prion-infected mice. JCI Insight. 2019 30;5. PMID: 31361599
